# Prevalence and endoscopic-histological correlation of premalignant gastric lesions at a university hospital in Uruguay

**DOI:** 10.1055/a-2542-0880

**Published:** 2025-03-14

**Authors:** Ignacio Moratorio, Adrian Canavesi, Carolina Olano, Klaus Mönkemüller

**Affiliations:** 1 226552 Unidad Académica Gastroenterología, Hospital de Clinicas Doctor Manuel Quintela, Montevideo, Uruguay

**Keywords:** Endoscopy Upper GI Tract, Precancerous conditions & cancerous lesions (displasia and cancer) stomach, Diagnosis and imaging (inc chromoendoscopy, NBI, iSCAN, FICE, CLE), Quality and logistical aspects, Training

## Abstract

**Background and study aims:**

Although chronic atrophic gastritis (CAG), intestinal metaplasia (IM), and dysplasia constitute gastric pre-neoplastic conditions of gastric cancer (GC), data on endoscopic correlation and the prevalence in many South American countries are scarce. The aims of this study were to establish prevalence and perform endoscopic-histological correlation of gastric pre-neoplastic conditions using high-definition white light endoscopy (WLE) and to determine interobserver agreement for endoscopic findings for CAG and IM.

**Patients and methods:**

A prospective, observational, descriptive, cross-sectional study was carried out at a Uruguayan hospital during a 6-month period. Risk was stratified according to Operative Link for Gastritis Assessment and Operative Link for Gastric Intestinal Metaplasia stage for CAG and IM, respectively. An independent and blinded second observer was included to determine interobserver endoscopic and histologic agreement.

**Results:**

A total of 102 patients (mean age 57 years ± 1.6 years, 68.6% woman) were included. Prevalence of histological CAG and IM were 38.2% and IM 31.4%, respectively. Endoscopic-histological correlation for CAG had kappa index 0.063, sensitivity 46%, and specificity 60%. For endoscopic IM, the kappa index was 0.216, sensitivity 22%, and specificity 96%. Interobserver variability was good for gastric fold flattening and very good in the presence of whitish-greyish plaques for CAG and IM, respectively.

**Conclusions:**

The endoscopic-histological correlation of both CAG and IM was low, raising the need for biopsy for diagnosis in all cases, regardless of HD-WLE findings. Although prevalence of gastric pre-neoplastic conditions in this group of Uruguayan patients was comparable to those described in countries with a high incidence of GC, a low proportion of high-risk stages (III and IV) was identified.

## Introduction

Gastric cancer (GC) is the fifth most frequent cancer in the world. Its distribution is heterogeneous with areas of high incidence such as East Asia, Eastern Europe, and Latin America 1 . In Western countries, diagnosis is usually accomplished in late stages, with involvement of the muscular layer of the stomach, leading to low rates of curative treatment and poor survival of less than 20% at 5 years 2 . In Uruguay, GC is the sixth cancer in incidence in men, but occupies fourth place in mortality 3 . Unlike Japan and South Korea, where successful screening programs for detection of gastric pre-neoplastic conditions and early GC have been successfully instituted, few other countries have followed suit 4 . Despite lack of screening programs, however, in some Western countries, upper endoscopy with biopsies and follow-up of patients with risk factors for GC is the standard method for early detection of pre-neoplastic conditions 5 .

Although the gold standard for diagnosis of gastric pre-neoplastic conditions such as chronic atrophic gastritis (CAG) and intestinal metaplasia (IM) is histology, there are endoscopic findings suggestive of them, such as visualization of submucosal vessels, flattening or loss of gastric folds for atrophy, as well as whitish-gray plaques or mottled patchy erythema and a tubular or tubulovillous mucosal pattern for IM 6 . According to the updated Sydney system, a total of five gastric biopsies—two from antrum, two from the corpus, and one from the incisura—are needed 7 . These must be collected in separated bottles by sectors, because that allows risk stratification according to the Operative Link on Gastritis Assessment (OLGA) and Operative Link on Gastritis/Intestinal Metaplasia Assessment (OLGIM) for CAG and IM, respectively 8 9 .

Histological correlation of pre-neoplastic conditions in the stomach with endoscopic findings is controversial and varies considerably between white light endoscopy (WLE) and use of advanced techniques such as magnification, digital chromoendoscopy, and laser confocal endomicroscopy 10 . These techniques have limitations such as high cost, increased study time, and issues with accessibility.

Depending on the extent and type of pre-neoplastic conditions in the stomach, together with the personal and family history of GC, surveillance of these patients is established. Early diagnosis of GC and its timely treatment have a survival rate of over 90% at 5 years. In contrast, the prognosis is ominous when it manifests clinically 2 .

The aims of this study were, first, to establish the prevalence of gastric pre-neoplastic conditions by histology, and second, to determine the ability of currently used WLE to diagnose gastric pre-neoplastic conditions, using histology as the gold standard. Lastly, we also determined interobserver agreement for endoscopic findings of CAG and IM.

## Patients and methods

This prospective, observational, descriptive, pilot, cross-sectional study was carried out at Hospital de Clínicas, Universidad de la República, Montevideo, Uruguay, which is a large university hospital for tertiary care.

All patients over age 18 years who underwent ambulatory upper endoscopy during the between June and November 2019 and who agreed to participate were included. Gastroesophageal reflux disease and dyspepsia were the main indications for gastroscopy. Patients with gastric resections, active gastrointestinal bleeding, coagulopathy, use of antibiotics or proton-pump inhibitors in the 14 days prior to the study, pregnant women, and those whose endoscopic findings were compatible with a digestive cancer were excluded. Prior to the endoscopic procedure, written informed consent was signed and clinical-epidemiological variables such as age, sex and *Helicobacter pylori* treatment were registered. Clinical and personal data were anonymized and handled confidentially.

All data generated or analyzed during this study are included in this article. Further enquiries can be directed to the corresponding author.

All procedures were performed by four endoscopists with more than 5 years of experience who conduct the procedures at the center and were experienced with high-definition WLE. In addition, before starting the recruitment phase of the study, all participants received training in recognition of endoscopic findings suggestive of *H. pylori* infection, CAG, IM, and early GC using endoscopic videos and photos. The Eluxeo 7000 system with series 600 gastroscopes and the Evis Exera II 180 system with series 150 and 160 gastroscopes were used for Fujifilm and Olympus systems, respectively. Although digital chromoendoscopy has been proven to enhance sensitivity and specificity for diagnosis of gastric pre-neoplastic conditions, it was not used in the procedures because not all endoscopes had this tool.

To determine interobserver agreement, an independent and blinded second group with advanced expertise in optical diagnosis of gastric pre-neoplastic conditions assessed presence or absence of defined criteria for CAG, IM, and dysplasia. Prior to biopsy collection, thorough and systematic characterization of the gastric mucosa was performed for approximately 6 minutes, with irrigation of water and simethicone in patients who presented with foam or bubbles. Gastric biopsies were performed on all patients, according to the modified Sydney protocol, with a total of five biopsies: two at the antrum (2–3 cm from the pylorus on the greater and lesser curvatures), one from the incisura, and two from the corpus (8 cm from the cardia on the greater and lesser curvatures). Antrum and incisura biopsies were collected in one jar and corpus biopsies in a separate jar. All samples were fixed in 10% buffered formalin and embedded in paraffin as routine procedure. Five-micron sections were made and stained with hematoxylin-eosin and Giemsa. All biopsies were assessed by a pathologist specialized in gastrointestinal pathology. Risk was stratified according to OLGA and OLGIM stage for histological CAG and IM, respectively. Analysis unit was per patient.

### Definitions

**Fig. 1:**
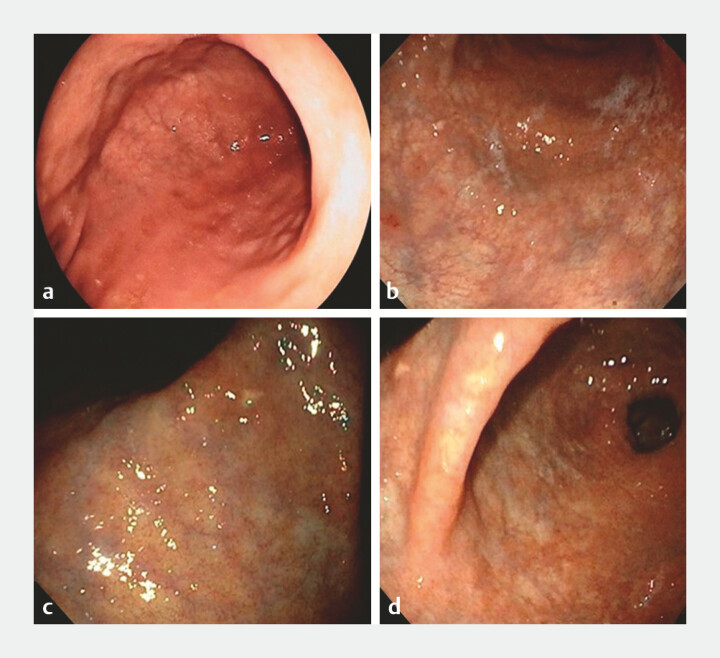
**a, b** Discoloration of the mucosa at the antrum with visualization of submucosal vessels. **c, d** chronic atrophic gastritis at the incisura.

**Fig. 2:**
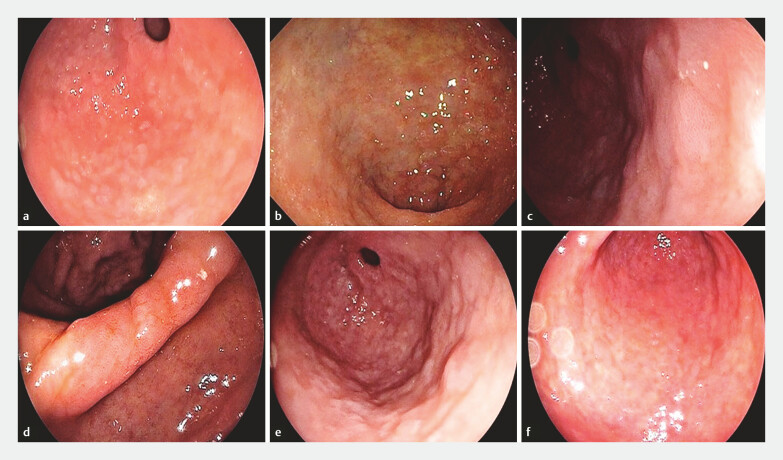
**a, b, c** Whitish-gray plaques at the antrum. **d** Whitish plaques on atrophic antral/incisura. **e** Intestinal metaplasia at the antrum and corpus. **f** Whitish plaques in the corpus.

Endoscopically, CAG was defined by presence of at least one of the following: discoloration of mucosa (pale color), flattening/loss of gastric folds, or thinning of mucosa with consequent visualization of submucosal vessels ( Fig. 1 ) 6 11 . IM was defined by presence of at least one of the following: patchy or homogeneous whitish-gray plaques or mottled patchy erythema ( Fig. 2 ) 11 .

Histologically, CAG was defined as a decrease in or loss of appropriate glands with expansion and replacement with fibrosis of the lamina propria and IM by loss of gastric epithelium replaced by different epithelium: intestinal or pseudopyloric 12 .

Histology was used as gold standard to determine CAG or IM. Therefore, prevalence of any gastric pre-neoplastic conditions was based on histological description of CAG or IM in at least one biopsy of any part of the sampled stomach (i.e. antrum, incisura, or corpus). Dysplasia was defined by unequivocal neoplastic changes of the gastric epithelium without evidence of stromal invasion 13 . It was categorized as low-grade dysplasia (LGD) (minimal architectural disorder and cellular atypia mild to moderate), high-grade (cuboid neoplastic cells, high nuclear-cytoplasmic ratio with amphophilic nucleoli, marked architectural disorder and numerous atypical mitoses), and undefined for dysplasia (presence of inflammatory component or artifacts that preclude diagnosis of dysplasia without characteristic findings of dysplasia) 14 . *H. pylori* was diagnosed based on direct microscopic observation of the microorganisms in gastric biopsies with hematoxylin and eosin and Giemsa staining. *H. pylori* -positive patients were referred to the Gastroenterology outpatient clinic, where the healthcare provider explained risks and benefits of treatment and the corresponding opportunity.

### Ethical aspects

The study protocol for this research was reviewed and approved by the Ethics Committee for Investigation from Hospital de Clínicas, Universidad de la República. The study was conducted following the ethical guidelines of Helsinki.

### Statistical analysis

A sample was used for convenience because the investigation corresponded to a pilot study, and thus, no sample calculation was performed. Frequency tables were presented to describe qualitative variables, as well as summary measures for continuous variables. Prevalence was calculated as the number of patients with the characteristic of interest per total number of patients at risk, expressed with a constant of 100. Study of the association between variables was performed with the chi-square test or Fisher's exact test in cases of expected values ​​less than 5. The difference for continuous variables between study groups was calculated with the Student's *t* -test after checking the normality of the variable with the Kolmogorov-Smirnov test. The concordance study between endoscopist and observer was performed with the Kappa index. In all cases, a *P* value of 0.05 was used. The software used corresponded to STATA v.12.0.

## Results

One hundred and two patients with a mean age of 57 years ± 1.6 years (range 18–87 years) were prospectively included. Seventy (68.6%) were women. Prevalence of *H. pylori* was 42% (43 patients). Table 1 shows patient clinical and demographic characteristics.

**Table:** **Table 1** Patients´ clinical and demographic characteristics

				Mean +/- SD	
Age (years)				56,9 ± 1,6	
**N (%)**					
Sex		Female		70 (68,6)	
		Male		32 (31,4)	
First-degree FH of GC				9 (8,8)	
*H. pylori* infection				43 (42)	
Eradication treatment effective against H. pylori.		YES		9 (69,2)	
		NO		4 (30,8)	
**PYI**					
Smoking or former smoker	NO		55 (53,9)	N/A	
	YES		47 (46,1)	Mild	18 (38,3)
				Moderate	11 (23,4)
				Severe	18 (38,3)
FH: familiy history. GC: gastric cancer. N/A: not applicable. PYI: Pack-year index; SD: standard deviation. N: number					

### Prevalence of gastric pre-neoplastic conditions

Prevalence of histological CAG and IM was 38.2% (39 patients) and 31.4% (32 patients), respectively.

According to OLGA risk stratification, 35 patients (34.3%) presented with low-risk stages (I-II) and 4 (3.9%) high-risk stages (III-IV).

According to OLGIM risk stratification, 29 patients (28.4%) presented with low-risk stages (I-II) and 3 (2.9%) high-risk stages (III-IV).

Only one patient (0.98%) presented with LGD and another undefined for dysplasia.

Prevalence of histological *H. pylori* was 42% (43 patients).

### Endoscopic findings

Diagnosis of endoscopic CAG or IM was suspected in 43 (42.2%) and 10 (9.8%) patients, respectively ( Table 2 , Table 3 , Fig. 1 and Fig. 2 ).

**Table:** **Table 2** Prevalence of endoscopic CAG and IM

	antrum/incisura N (%)	corpus N (%)	total N (%)
CAG	42 (41.2%)	12 (11.8%)	43 (42.2%)
IM	8 (7.8%)	4 (3.9%)	10 (9.8%)
CAG: chronic atrophic gastritis; IM: intestinal metaplasia			

**Table:** **Table 3** Endoscopic findings for the diagnosis of CAG and IM

	CAG	N (%)	IM	N (%)
Findings in antrum/incisura	Mucosal discoloration	36 (35.3)	Whitish-gray plaques	6 (5.9)
	Mucosal thinning	32 (31.4)	Mottled patchy erythema	2 (1.9)
	Increased light refractoriness	25 (24.5)		
	Visualization of submucosal vessels	31 (30.4)		
Findings in corpus	Mucosal discoloration	4 (3.9)	Whitish-gray plaques	2 (1.9)
	Mucosal thinning	5 (4.9)	Mottled patchy erythema	2 (1.9)
	Increased light refractoriness	4 (3.9)		
	Visualization of submucosal vessels	6 (5.9)		
	Flattening of gastric folds	9 (8.8)		
	Loss of gastric folds	7 (6.9)		
CAG: chronic atrophic gastritis; IM: intestinal metaplasia; N: number.				

### Endoscopic-histological correlation

Eighteen of 43 patients who presented with endoscopic appearance of CAG had histological CAG according to the OLGA stage ( Table 4 ) Strength of agreement was poor, kappa index of 0.063 ( Table 5 ). Sensitivity was 46% and specificity 60%. The positive predictive value (PPV) was 42% and the negative predictive value (NPV) 64%.

**Table:** **Table 4** Endoscopic-histological correlation of CAG

ENDOSCOPY		OLGA STAGE					Total
		0	I	II	III	IV	
CAG	PRESENCE	25	12	3	3	0	43
	ABSENCE	38	13	7	0	1	59
CAG: chronic atrophic gastritis; OLGA: operating system Link on Gastritis Assessment;							

**Table:** **Table 5** Interobserver Agreement for CAG and IM

Findings antrum/incisura and/or corpus	Kappa Index	Strength
Mucosal discoloration	0.248	poor
Mucosal thinning	0.458	moderate
Increased light reflection	0.439	moderate
Visualization of submucosal vessels	0.512	moderate
Flattening of gastric folds	0.694	good
Loss of gastric folds	0.580	moderate
Whitish-gray plaques	0.918	very good
Mottled patchy erythema	0.682	good
CAG: chronic atrophic gastritis; IM: intestinal metaplasia		

For IM, only 10 patients were identified with endoscopy, whereas according to OLGIM stage, 32 presented with histological IM ( Table 6 ). Strength of agreement was weak, kappa index of 0.216 ( Table 5 ). Sensitivity was 22% and specificity 96%. PPV was 70% and NPV 73%.

**Table:** **Table 6** Endoscopic-histological correlation of IM

ENDOSCOPY		OLGIM STAGE					Total
		0	I	II	III	IV	
IM	PRESENCE	3	4	1	2	–	10
	ABSENCE	67	21	2	2	–	92
IM: intestinal metaplasia; OLGIM: operative Link on Gastritis/Intestinal-Metaplasia Assessment.							

## Discussion

In this study, prevalence of a histological gastric pre-neoplastic condition was 38.2% for CAG and 31.4% for IM. In contrast, prevalence of dysplasia or cancer was very low, at 1%.

It is interesting that prevalence of histological CAG found in this study was similar to that in Latin American countries with a high incidence of GC (Colombia 37%-42% and Peru 35%) 15 16 17 18 . However, for histological IM, prevalence was similar to a Turkish publication (31%) with high incidence of GC 19 .

Surprisingly, overall prevalence of gastric pre-neoplastic conditions found in this Uruguayan study was significantly higher than those reported in South American countries with similar incidence rates for GC. Whereas in Brazil incidence of GC is similar to Uruguay, prevalence of histological CAG (10%) and IM (14%) were much lower than ours 20 . In addition, two studies from Argentina showed that prevalence of histological IM was 7.9% and 11% respectively 21 . It is possible that differences in patient populations seen at the hospital may explain these differences. However, demographics and hospital settings in the Argentinian and Brazilian studies are quite similar to ours. Although H. pylori infection is considered the main risk factor for histological progression 22 , differing prevalence of gastric pre-neoplastic conditions may not only be influenced by H. pylori because prevalence of the H. pylori infection is similar in Brazil, Argentina, and Uruguay 23 24 . More virulent H. pylori strains and/or other risks factors such as advanced age, tobacco use, and family history of GC may be responsible for histological presence and/or progression of CAG and IM. Furthermore, differences in microbiota composition may be one of the major factors contributing to progression of gastric pre-neoplastic conditions to cancer, as has recently been shown in a Colombian study 25 . Colombians living in the Andes mountains have H. pylori infection rates similar to those living in coastal areas. However, GC incidence is 25 times higher than in the population living in coastal areas 26 .

Despite the high prevalence of gastric pre-neoplastic conditions found in this study, prevalence of advanced stages (OLGA and OLGIM III-IV) and dysplasia was low. These results differ from those in Eastern countries, where prevalence of advanced stages is high, as reported in the study by Satoh et al 9 . Similarly, prevalence of advanced stages of CAG was also lower in this study than in Latin American countries with a high prevalence of GC (Colombia and Peru) 15 16 17 .

Prevalence of histological dysplasia identified in this study was 0.98% (1 patient), which coincides with global prevalence, which varies from 0.5 to 3.8% in Western countries but much less than in areas with a high incidence of GC (9% to 20%) 27 . This gastric pre-neoplastic condition corresponded to LGD and was found in a patient with stage III on the OLGA and OLGIM systems. Nevertheless, based on the high prevalence of pre-neoplastic conditions in the stomach such as CGA and IM, prevalence of advanced lesions or cancer should have been higher.

Another notable aspect of our study was the weak endoscopic-histological correlation for CAG and IM using HD-WLE: 43 vs 39 patients and 10 vs 32, respectively. Although some authors affirm that detection of pre-neoplastic conditions can be performed after detailed analysis of the gastric mucosa with HD-WLE 6 28 29 , others argue that diagnosis cannot be reliably made with this modality and other techniques, such as magnification chromoendoscopy or narrow-band imaging, are required for accurate endoscopic diagnosis 28 29 30 31 .

According to a study that evaluated interobserver variability and diagnostic accuracy of endoscopic IM between experienced and unexperienced endoscopists, agreement for both groups was poor (Kappa index 0.38 and 0.33, respectively), whereas diagnostic accuracy was statistically higher for experienced groups 32 . In contrast, in this study, interobserver strength of agreement identified for presence of whitish-grey plaques and mottled erythema in the stomach was good and very good between the endoscopist and the observer (kappa index 0.68 and 0.92 respectively). This may be due to researchers’ heightened level of suspicion, as well as prior training in recognizing these findings. Similarly, the authors emphasize the need for endoscopic education and training to increase diagnostic accuracy of IM, as well as taking biopsies of any area on endoscopy considered suspicious for ​​IM 32 . Lim et al. found similar results and concluded that a high index of suspicion is important to increase sensitivity of endoscopic diagnoses of IM, and histological confirmation is necessary 33 .

This study has important strengths. First, it is the first to evaluate prevalence of gastric pre-neoplastic conditions in a South American country, specifically Uruguay, adding valuable data to the literature on the topic and allowing for comparisons with other studies in the region. Second, we performed a careful endoscopic-histological correlation, as well as assessment of interobserver agreement for endoscopic findings of CAG and IM in a real-world clinical practice setting. Furthermore, our study differs from previous ones in that interobserver agreement was conducted in situ, during the same procedure, using live video imaging. Most studies evaluating interobserver agreement rely on still images or selected or edited video clips, which do not accurately reflect real practice.

We want to highlight potential limitations. We are a tertiary care hospital and the population or staff performing the procedure may not represent other clinical settings. However, our hospital also provides primary care ambulatory endoscopy, thus balancing this effect. Second, the sample size was relatively small. Nevertheless, the data were collected prospectively and consecutively, diminishing the chance of bias.

## Conclusions

In summary, we found that prevalence of gastric pre-neoplastic conditions in the group of Uruguayan patients that was studied was high. Although this is comparable to countries with a high incidence of GC, in our population, we identified a much lower proportion of high-risk stages (III and IV) and dysplasia or cancer. This raises the possibility that other factors in addition to *H. pylori* must play a role in development and progression of GC. Lastly, we found that endoscopic-histological correlation of both CAG and IM was poor, which dictates the need for histologic diagnosis in all cases, regardless of endoscopic findings identified with HD-WLE.
